# Safety and Efficacy of Devices Delivering Inhaled Antibiotics among Adults with Non-Cystic Fibrosis Bronchiectasis: A Systematic Review and a Network Meta-Analysis

**DOI:** 10.3390/antibiotics11020275

**Published:** 2022-02-19

**Authors:** Sofia Tejada, Sergio Ramírez-Estrada, Carlos G. Forero, Miguel Gallego, Joan B. Soriano, Pablo A. Cardinal-Fernández, Stephan Ehrmann, Jordi Rello

**Affiliations:** 1 Clinical Research/Epidemiology in Pneumonia & Sepsis (CRIPS), Vall d’Hebron Institut of Research (VHIR), 08035 Barcelona, Spain; jrello@crips.es; 2 Centro de Investigación Biomédica En Red de Enfermedades Respiratorias (CIBERES), Instituto de Salud Carlos III, 28029 Madrid, Spain; mgallego@tauli.cat (M.G.); jbsoriano2@gmail.com (J.B.S.); 3 Intensive Care Department, Clínica Corachán, 08017 Barcelona, Spain; sergioramirezestrada@hotmail.com; 4 School of Medicine, Universitat Internacional de Catalunya, 08195 Barcelona, Spain; cgarciaf@uic.es; 5 Respiratory Department, Parc Taulí University Hospital, 08208 Barcelona, Spain; 6 Hospital Universitario La Princesa, Universidad Autónoma de Madrid, 28006 Madrid, Spain; 7 Intensive Care Unit, HM Group, 28015 Madrid, Spain; pablocardinal@hotmail.com; 8 CHRU Tours, Médecine Intensive Réanimation, CIC INSERM 1415, CRICS-TriggerSEP F-CRIN Research Network, 37000 Tours, France; stephanehrmann@gmail.com; 9 Centre d’étude des Pathologies Respiratoires, INSERM U1100, Université de Tours, 37032 Tours, France; 10 Clinical Research in the ICU, CHU Nimes, Universite de Nimes-Montpellier, 30900 Nimes, France; 11 Medicine Department, Universitat Internacional de Catalunya (UIC), 08195 Sant Cugat, Spain

**Keywords:** bronchiectasis, non-cystic fibrosis, dry powder inhaled, small-volume nebulizer, inhaled antibiotics

## Abstract

It remains unknown whether the type of aerosol generating device is affecting efficacy and safety among non-cystic fibrosis bronchiectasis (NCFB) adults. The proposal of this network meta-analysis (NMA) is to evaluate effectiveness and safety of inhaled antibiotics administered via dry powder inhaler (DPI) and via nebulizers (SVN) among adult patients with NCFB. Inclusion criteria were randomized-controlled trials, adults (≥18 years) with NCFB, and inhaled antibiotics administered via DPI as intervention. Search strategy was performed in PubMed, Web of Science, and Cochrane Library from 2000 to 2019. Sixteen trials (2870 patients) were included. Three trials (all ciprofloxacin) used DPIs and thirteen used SVN (three ciprofloxacin). Both DPI and SVN devices achieved similar safety outcomes (adverse events, antibiotic discontinuation, severe adverse events, and bronchospasm). Administration of ciprofloxacin via DPI significantly improved time to first exacerbation (87 days, 95% CI 34.3–139.7) and quality of life (MD −7.52; 95% CI −13.06 to −1.98) when compared with via SVN. No other significant differences were documented in clinical efficacy (at least one exacerbation, FEV_1_% predicted) and microbiologic response (bacterial eradication, emergence of new potential pathogens, and emergence of antimicrobial resistance) when comparing devices. Our NMA documented that time to first exacerbation and quality of life, were more favorable for DPIs. Decisions on the choice of devices should incorporate these findings plus other criteria, such as simplicity, costs or maintenance requirements.

## 1. Introduction

Inhaled antibiotics have been used to treat bronchial colonization/infection, especially in cystic fibrosis patients with chronic bronchial infection by *Pseudomonas aeruginosa* [1]. Although most experts agree on the positive effects, and international clinical guidelines recommended their use [2,3,4,5], the role of inhaled antibiotics as first-line therapy among non-cystic fibrosis bronchiectasis (NCFB) remains controversial [6].

Devices used to deliver therapeutic agents as aerosols are based on nebulizers (SVN), pressurized metered-dose inhalers, or dry powder inhalers (DPI) [7], mostly for beta-agonists, anti-cholinergic agents, and steroids [8]. Clinical insight suggests that not only different types of devices, but even different models of the same device, can make a difference on efficiency [9].

There are established indications on administering inhaled antibiotics mainly in children or young adults with cystic fibrosis. Their use has been extended to mechanical ventilated [10] and NCFB adult patients, mainly for acute or chronic *P. aeruginosa* infection [6,11,12,13]. Most of these studies were conducted with SVN devices. In out-of-hospital patients with bronchiectasis, DPIs were introduced a decade ago, but questions remain concerning if they are as clinically effective, safe and cost effective as nebulized antibiotics [14]. Clinical practice guidelines (CPG) do not provide recommendations on their use, and it remains unknown whether the type of aerosol generating device is affecting efficacy and safety among NCFB adults. Whereas most studies tested SVN [9], quicker and more convenient formulations of antipseudomonal antibiotics have been recently developed in the form of DPI.

Several systematic reviews with traditional pairwise meta-analysis of randomized-controlled trials (RCT) have assessed the effectiveness of inhaled antibiotics versus placebo in NCFB patients [6,11,12]. However, this approach is not suitable for comparing devices, and RCTs with all device options are not available. Network meta-analysis (NMA) is a statistical tool that allows the analysis of the simultaneous comparison between interventions from different studies. In addition, it enables sorting all interventions according to their probability of being the best, even when they have never been compared in a face to face study [15]. Recently, NMAs have been applied to several diseases and their conclusions have been considered in recognized CPGs to increase the level of recommendations. However, NMA has never been applied to inhale antibiotic devices.

We hypothesize that there are no differences in terms of efficacy depending on the medication and type of administration (SVN, DPI). To this end, this NMA answers the following question: Are inhaled antibiotics administered by different devices similar in terms of clinical, microbiological and safety outcomes in adult patients with bronchiectasis without cystic fibrosis?

## 2. Results

### 2.1. Study Selection

**Figure 1:**
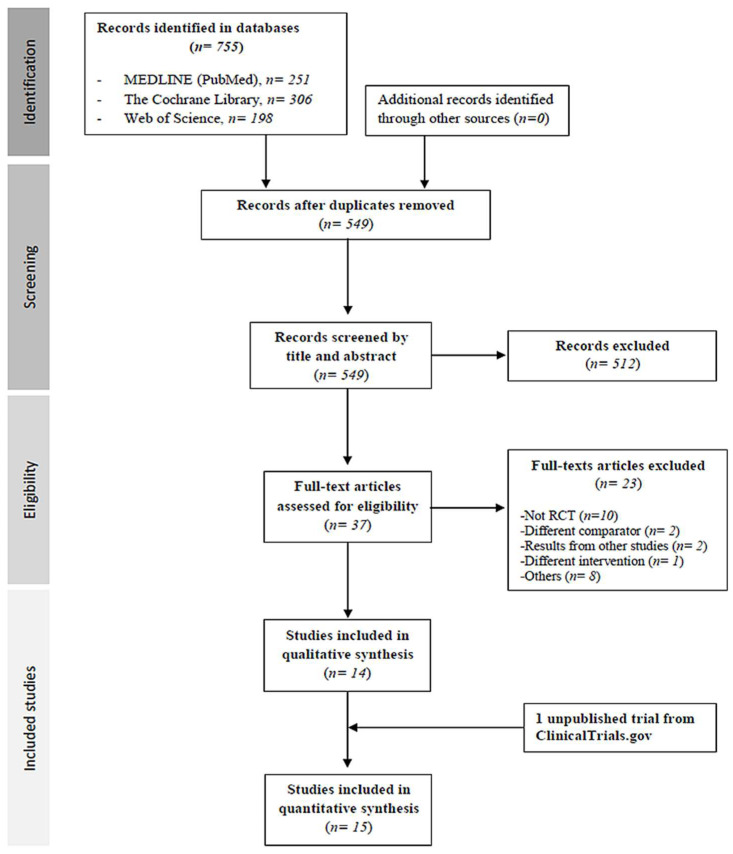
PRISMA flow diagram of the study selection process in the analysis.

The search identified 755 potentially relevant studies. After applying the inclusion and exclusion criteria, 15 articles [16,17,18,19,20,21,22,23,24,25,26,27,28,29,30] (with 16 independent RCTs) were finally included in our meta-analysis. Reasons for exclusion are detailed in Table S1. Two articles contained two RCTs [19,21]. One RCT was reported both by Barker et al. in 2000 and Couch et al. in 2001, so these RCTs were labelled as Barker/Couch [28,29]. Flowchart process is shown in Figure 1.

### 2.2. Study Characteristics

A total of 16 RCTs, recruiting 2870 patients, were included. Among them, 2294 (80%) patients had NCFB and *P. aeruginosa* chronic bronchial infection. All were stable patients at baseline. Six RCTs had >2 exacerbations requiring antibiotic therapy within 12 months and the other ten were not reported. The mean age of patients treated with DPI and SVN devices were 62 and 61.1, respectively. Population characteristics are detailed in Table S2.

All DPI trials administered ciprofloxacin [16,17,18]. Three trials (from two studies [19,24]) administered ciprofloxacin via SVN. Ten more trials administered inhaled antibiotics via SVN: four tobramycin (from five studies [22,26,27,28,29]), two amikacin [20,30], two aztreonam (from one study [21]), one gentamycin [25], and one colistin [23]. Study characteristics are detailed in Table 1. The comparator was always a placebo. Studies of inhaled antibiotics comparing DPI to SVN among NCFB were not found.

### 2.3. Risk of Bias Assessment

Six of fifteen RCTs showed a low risk of bias in all Cochrane tool domains (Figure S1). Nine RCTs showed at least one high risk of bias. One trial was unpublished and could not be fully evaluated [30].

### 2.4. Outcomes

#### 2.4.1. Efficacy

**Figure 2:**
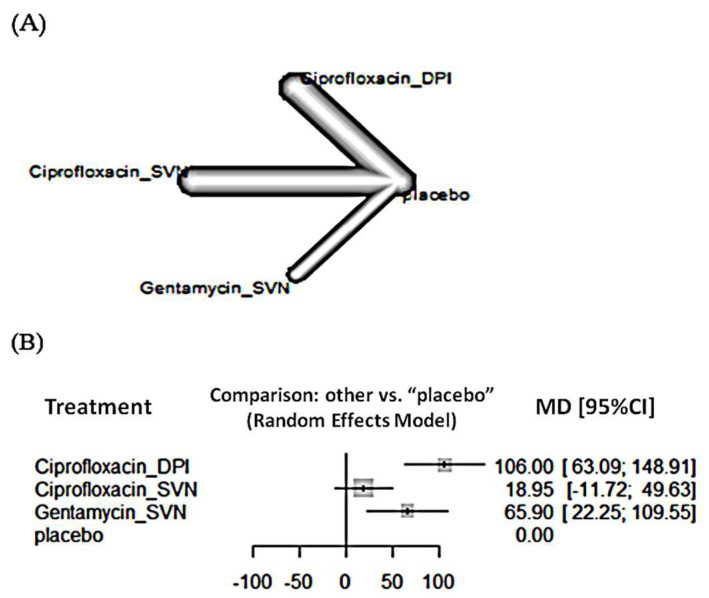
(**A**) Network plot and (**B**) forest plot of the time to first exacerbation.

For time to first exacerbation, the network included seven RCTs and 8379 patients. Based on interval estimation of direct and indirect comparison, ciprofloxacin via DPI significantly increased time to first exacerbation in 87 days (95% confidence interval [CI] 34.30–139.79) when compared with ciprofloxacin via SVN. The treatment with the highest probability of being the best is ciprofloxacin administered via DPI (Figure 2). 

A total of 38.9% (266/683) of patients receiving inhaled antibiotics via DPI and 42.4% (358/844) of patients via SVN (*p* = 0.17) experienced at least one exacerbation. The treatments with the highest probability of being the best are gentamycin and colistin via SVN followed by ciprofloxacin via DPI (Figure S2). There were no statistical differences between ciprofloxacin devices with an NNT of 28 (Table 2). Funnel plots is reported in Figure S3.

Based on interval estimation of direct and indirect comparison, ciprofloxacin via DPI improves quality of life as St. George’s Respiratory Questionnaire (Mean Difference [MD] −7.52; 95% CI −13.06 to −1.98) when compared to via SVN (Table 2). No significant improvement of either spirometry as forced expiratory volume in one second (FEV1%) was reported. Quality of life and spirometry results are shown in Figure S2. Funnel plot is reported in Figure S3.

#### 2.4.2. Microbiological Outcomes

**Figure 3:**
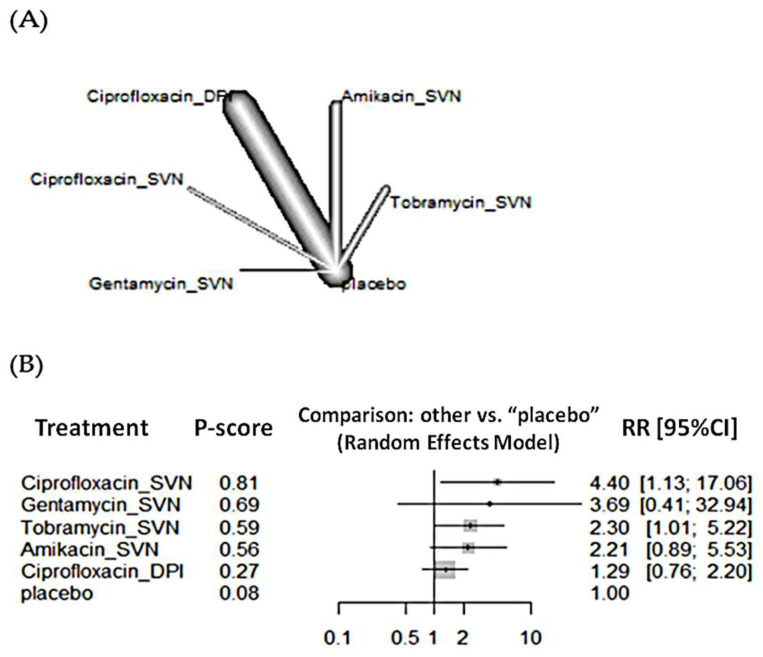
(**A**) Network plot and (**B**) forest plot of bacterial eradication.

For bacterial eradication, the network included nine RCTs and 1351 patients. The treatment with the highest probability of being the best is ciprofloxacin administered via SVN (Figure 3). Administered ciprofloxacin via SVN significantly increased bacterial eradication when compared to placebo (Relative Risk [RR] 4.40, 95% CI 1.13–17.06) but not compared to ciprofloxacin via DPI (Table 2). Sputum bacterial load was not significantly different when comparing ciprofloxacin devices (Table 2 and Figure S2). Funnel plot is reported in Figure S3.

For emergence of new potential pathogens, the network included five RCTs and 1131 patients. The treatment with the highest probability of being the best is ciprofloxacin administered via DPI (Figure S2). When compared with placebo, ciprofloxacin via DPI significantly reduced new potential pathogens (RR 0.48, 95% CI 0.31–0.74) with an NNT of 11. There were no statistically significant differences between devices (Table 2). Funnel plot is reported in Figure S3.

**Figure 4:**
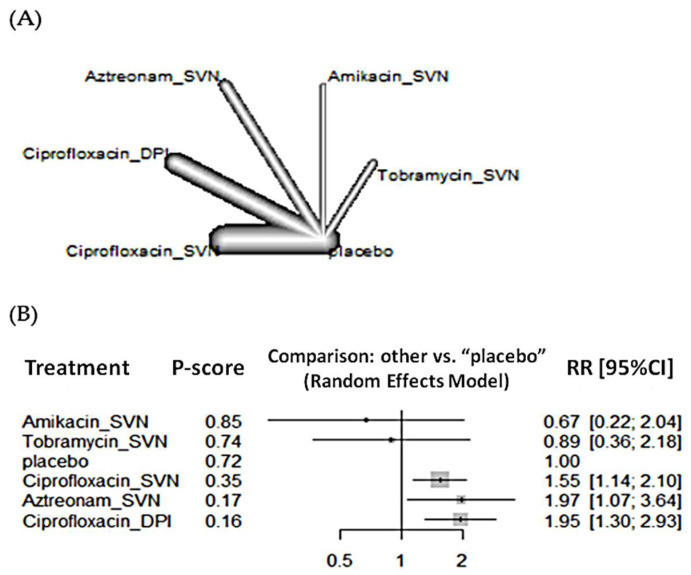
(**A**) Network plot and (**B**) forest plot of emergence of antimicrobial resistance.

For emergence of antimicrobial resistance, the network included 12 RCTs and 1905 patients. The treatment with the highest probability of being the best is amikacin followed by tobramycin, and ciprofloxacin administered via SVN (Figure 4). Administration of ciprofloxacin via DPI significantly increased antibiotic resistance when compared to placebo (RR 1.95, 95% CI 1.30–2.93), but there were no statistically significant differences between devices (Table 2). No statistically significant differences to reduce the emergence of *P. aeruginosa* resistance were observed (Table 2 and Figure S2).

#### 2.4.3. Mortality

Mortality rates were low across all trials (1.7% using DPI vs. 2.1% using SVN, *p* = 0.62) and no treatment seemed to be superior to others. Treatments with the highest probability of being the best are colistin and ciprofloxacin via SVN (Table 2 and Figure S2).

#### 2.4.4. Safety Outcomes

No significant differences between devices in all safety outcomes were found, although ciprofloxacin administered via DPI has a higher probability of being better than ciprofloxacin administered via SVN (Table 2 and Figure S2).

## 3. Discussion

This study is the first to compare safety and effectiveness of inhaled antibiotics in non-hospitalized adults with NCFB, depending on the aerosol generating devices. Both DPI and SVN devices have comparable benefits in terms of clinical resolution or mortality rates, and both induced a significant risk of antibiotic resistant bacteria acquisition. Adverse events were minimal, but no improvement in safety outcomes was documented. However, some indicators of clinical resolution favored the use of DPIs, translating into a delay (estimated in 87 days) of the time to first exacerbation and improving quality of life. The effectiveness of delivery devices may provide a basis for selecting one device over another.

The main objective of inhaled antibiotics is to deliver a sufficient amount of antibiotics into airways. To achieve this goal, patient-related factors and particle-related factors are critical to bypass the upper airways [31]. Due to the lack of studies in NCFB, most evidence comes from the cystic fibrosis population, especially with tobramycin and colistin based care [32,33]. In a randomized study undertaken in cystic fibrosis patients comparing tobramycin via DPI vs. nebulized tobramycin, effectiveness and adverse events were similar in both groups. Study-related cough was reported as an adverse event in 25% of subjects with DPI versus 4% of the subjects on SVN [33], although in most cases this did not require medication withdrawal. In a real-world study performed in 164 NCFB patients, who were treated with colistin or tobramycin via DPI [7], 24.4% of them were withdrawn from treatment, mainly due to cough. Main risk factors were previous coughing, COPD and insufficient patient instruction regarding how to use the device. In our meta-analysis, when comparing devices adjusted by antibiotic class, no differences were identified. One point to consider is that the adverse events were probably recorded differently across studies. Drug delivery may be improved through sustained-release formulations, new inhaler technologies, and optimization of aerosol properties of DPI formulations.

Our findings suggest that mortality rates or clinical efficacy do not justify a clear preference for one device. However, devices have different microbiologic responses, translating in a significant delay in time to first pulmonary exacerbation. In essence, our findings suggest that each of the devices can work well if patients use them appropriately, with small advantages favoring DPIs. This is an important observation, because in the outpatient setting the selected patients were trained to use the appropriate technique. The trials included in this systematic review do not provide information regarding who is more likely to use one device versus other, including patient references, or the ability of using the device, equipment availability and costs. Whilst DPIs are anticipated to improve patient adherence because of ease of administration and increased convenience, other considerations such as cost effectiveness need to be considered. The DPIs are more portable than nebulizers and are also simpler and quicker to prepare and administer. Patients should not be spending hours preparing, using and cleaning nebulizers for inhaled antibiotics.

Optimizing aerosolization is key to improving safety and clinical efficacy, which is easy to set up. An excellent meta-analysis [34] on aerosolized antibiotics in acute lung infection has been recently reported. Comprehensive position papers or guidelines for aerosol therapy are available in asthma [9], or antibiotics for healthcare and ventilator-associated pneumonia [35,36]. Specific position papers on devices in ventilated patients are available [10]. Unfortunately, their recommendations cannot be translated to NCFB. A recent review [37] assessed the quality of clinical practice guidelines for aerosolization of antibiotics in NCFB using the AGREE II tool. Whereas recent guidelines have improved the quality of recommendations, particularly on antibiotic therapy, no information was reported regarding device selection. Our study suggests that the use of a microbiological endpoint such as bacterial eradication is questionable, and most trials were associated with selection of resistant flora. Thus, further larger trials using DPI are needed. Furthermore, a comparison with a non-aerosol strategy such as cycling rotation of macrolides for prevention or oral macrolides for therapy of acute exacerbations in chronic airway infections [33,38] is required. The role of the airway’s microbiota is instrumental and the differential conditions for therapy of chronic (rather than acute airway) infection need to be considered, with immune and inflammatory response playing an important role [39]. Thus, a new paradigm addressing dysbiosis is required [39]. Evidence-based guidelines to assist in the selection of different aerosol delivery devices for NCFB are an unmet clinical need. Current NMA would be of help it. When selecting an aerosol delivery device for antibiotics among patients with NCFB, several factors need to be considered (Table 3). The outpatient setting has specific conditions and patient preferences need to be considered.

It is clear that proper patient education is critical, whichever device is chosen, and assessment of technique of inhalation should be submitted to follow up. Nurses, respiratory therapists, and physicians caring for bronchiectasis adults should be familiar with the correct use of the device and its performance. Patients have to be instructed in the right use of aerosol delivery devices. If the selected device results in unacceptable adverse events or fails to provide satisfactory outcomes, both patients and clinicians should be aware of other effective strategies, such as cycling macrolides [38,40].

### Strengths and Limitations

This NMA has some limitations. The main limitation is the small sample size of DPI (all trials of ciprofloxacin) precluding many subgroup comparisons. Additional large trials of inhaled antibiotics administered by DPI compared with SVN devices are needed, to explore potential differences in events with small prevalence, such as bronchospasm. Moreover, no studies comparing DPI vs. SVN were documented, and only RCTs comparing interventions versus placebo were identified. Due to the lack of studies comparing DPI vs. SVN, our analyses provide added value. Trial designs were heterogeneous in terms of the endpoints used, the duration of studies, the choice of inhaled antibiotics, and differences in drug dose between DPI and SVN. In addition, the adverse events were recorded differently across studies. Although we recorded a large amount of information, data on many endpoints were incomplete because they were reported in a format that could not be extracted and assessed. Studies have been largely unselective in terms of cause of bronchiectasis, severity of disease, lung function, and concomitant therapy. Strengths of this NMA include the restricted design to RCT, hypothesis comparing DPI vs. SVN devices, comprehensive search, pre-registration of the protocol, careful assessment of subgroups of interest, risk of bias assessment using RevMan 5.3, and performed meta-analysis to evaluate the impact of inhaled antibiotics with the different devices using R software.

## 4. Methods

### 4.1. Registration and Protocol

This study was performed following the Preferred Reporting Items for Systematic Reviews and Meta-analysis for Network-Met-analysis (PRISMA-NMA) guidelines [41,42], and followed the recommendations of the Cochrane Handbook for Systematic Reviews of Interventions [43]. The PRISMA-NMA checklist is reported in Table S3. The protocol was registered on PROSPERO (CRD42021253700).

### 4.2. Eligibility Criteria

We used the following inclusion criteria: (i) RCT; (ii) adults (≥18 years) with NCFB; (iii) inhaled antibiotics administered via DPI at any dose as intervention. Antibiotics included were ciprofloxacin, tobramycin, amikacin, aztreonam, gentamycin, and colistin. Devices’ definitions were reported elsewhere [44].

The following outcomes of interest were analyzed:

- *Clinical efficacy:* time to fist exacerbation, number of patients at least one exacerbation, spirometry results as FEV1%, and quality of life measured by SGRQ. In this SGRQ questionnaire, higher scores indicate a poorer quality of life.
- *Clinical outcomes:* mortality.
- *Microbiological outcomes:* bacterial eradication, emergence of new potential respiratory pathogens, sputum bacterial density, and emergence of overall (and *P. aeruginosa*) antimicrobial resistance.
- *Safety outcomes:* adverse events related to study drug, serious adverse events, adverse events leading to study drug discontinuation, and bronchospasm episodes.

Outcome definitions are detailed in Table S4. Exacerbation definition was reported elsewhere [6].

### 4.3. Search Strategy

A global search strategy was systematically performed in PubMed, Cochrane Library Database, and Web of Science database. We also searched the ClinicalTrials.gov and clinicaltrialsregister.eu registers to identify ongoing trials.

A restriction was also applied to the publication time period, limiting it from 2000 to 2019 aimed at focusing on a reflection of current care practices. Literature search was limited to human subjects. No language restrictions were applied. Search strategy is detailed in Table S5. To ensure literature saturation, we scanned the reference lists of included studies, relevant reviews, or previous systematic review and meta-analysis identified through the search [11,12]. Studies regarding to patients with cystic fibrosis were excluded.

### 4.4. Data Collection

Two reviewers (S.T. and S.R.E.) independently screened the titles, abstracts, and full-text yielded by the search against the inclusion criteria. A third reviewer (J.B.S.) adjudicated disagreements, when necessary. A standardized form in Excel to collect data was performed. Data abstracted included patient characteristics, trial characteristics, type of intervention and comparator (dosage, frequency and duration of treatment), and outcomes extracted.

### 4.5. Quality Assessment

Two authors (S.T. and C.G.F.) independently assessed the risk of bias using the Cochrane’s tool for assessing risk of bias. Disagreement regarding quality assessment was resolved by a third author (J.R.). Seven aspects were included as follows: random sequence generation (selection bias), allocation concealment (selection bias), blinding of participants and investigators (performance bias), blinding of outcome assessment (detection bias), incomplete outcome data (attrition bias), selective reporting (reporting bias), and other bias. Each of the components was classified as “yes”, “unclear”, or “no”, which represent “low risk of bias”, “unclear risk of bias”, and “high risk of bias”, respectively. A study was considered with low risk of bias when all their domains were classified as low risk. Review Manager Software (version 5.3) was used to assess the validity of studies included.

### 4.6. Statistical Analysis

For dichotomous outcomes, the number of patients with each outcome and denominator were extracted. For continuous outcomes, sample size, mean (standard deviation) or median (interquartile range) were extracted, based on the information provided in their respective publications. Continuous variables reported as percentiles were transformed in mean and standard [45]. The mean difference between groups for the continuous variables was computed to compare the effect of treatments.

The dichotomous outcome was expressed as RR with 95% CI and continuous outcomes was expressed as MD with 95% CI. Placebo was always the reference treatment. Random effects model was used assuming heterogeneity across the studies. The overall inconsistency was assessed by the I^2^ statistics; it is imprecise with 95% CIs.

We used network plots to illustrate the map of the direct and indirect comparisons. The results of the meta-analyses were reported in a forest plots. Matrix tables were used to report the results of direct and indirect comparisons. Network geometry was qualitatively described [46]. The probability of being the best intervention was calculated using the P-score method. This procedure is analogous to the surface under the cumulative ranking curve (SUCRA) method and is based solely on the point estimates and standard errors of the frequentist NMA estimates under normality assumption. This means that, if treatment c is better than treatment a, b and d, the P-score of treatment c will be higher than the others. In other words, the higher the treatment’s P-score, the higher the probability of it being the best.

Funnel plots together with Egger test were used to assess publication bias, if it existed.

A *p*-value < 0.05 was considered statistically significant. The network meta-analysis was performed using the frequent method thought the *netmeta* library of the R software version 4.0.3.

## 5. Conclusions

When delivering inhaled antibiotics among adults with NCFB, ciprofloxacin via DPI was non-inferior to SVN in clinical efficacy, safety, microbiologic response, and mortality. Ciprofloxacin via DPI significantly delayed time to first exacerbation. Decisions to choose devices should incorporate these findings plus other criteria, such as simplicity, costs or maintenance requirements.

## Figures and Tables

**Table 1:** Characteristics of randomized-controlled trials included in the meta-analysis, stratified by devices.

Study	Year	Location	Age, Mean (SD)	Characteristics	N Patients	Intervention	Control	Doses	Frequency
***DPI device***									
deSoyza (RESPIRE-1)	2018	Israel, Australia, New Zealand, Spain, USA, UK, Germany, Japan, Italy, Latvia, France, Argentina, Slovakia, Denmark	Ciprofloxacin: 64.2 (12.1) Placebo: 64 (13.5)	RDBPCT, multicenter	416	Ciprofloxacin	Placebo	32.5 mg	BID, 48 weeks of 14 or 28 days on/off via DPI
Aksamit (RESPIRE-2)	2018	Russia, Bulgaria, Latvia, Poland, South Korea, Serbia, Romania, Turkey, Australia, Germany, The Netherlands, USA, Brazil, Portugal, China, Austria, Argentina, Thailand, Taiwan, South Africa, Philippines, Lithuania, Czech Republic	Ciprofloxacin: 59.3 (14.2) Placebo: 60.6 (13.7)	RDBPCT, multicenter	521	Ciprofloxacin	Placebo	32.5 mg	BID, 48 weeks of 14 or 28 days on/off via DPI
Wilson	2013	Australia, Germany, Spain, Sweden, UK, USA	Ciprofloxacin: 64.7 (11.8) Placebo: 61.4 (11.9)	RDBPCT, multicenter	124	Ciprofloxacin	Placebo	32.5 mg	BID, 28 days on and 56 weeks off via DPI
***SVN device***									
Haworth (ORBIT 3)	2019	Australia, Canada, Germany, Hungary, Ireland, Israel, Italy, Latvia, Poland, UK, USA, Romania, South Africa, South Korea, Spain, Taiwan	Ciprofloxacin: 64.3 (13.6) Placebo: 66.7 (10.7)	RDBPCT, multicenter	278	Ciprofloxacin ARD-3150	Placebo	6 mL (3 mL liposome- encapsulated ciprofloxacin 135 mg + 3 mL free ciprofloxacin 54 mg)	QD, 48 weeks (6 cycles) of 28 day on/off via nebulizer (PARI LC sprint)
Haworth (ORBIT 4)	2019	Australia, Canada, France, Georgia, Hungary, Israel, Italy, New Zealand, Peru, Poland, Romania, Serbia, South Korea, Spain, UK, USA	Ciprofloxacin: 63.3 (13.5) Placebo: 64.2 (12.6)	RDBPCT, multicenter	304	Ciprofloxacin ARD-3150	Placebo	6 mL (3 mL liposome- encapsulated ciprofloxacin 135 mg + 3 mL free ciprofloxacin 54 mg)	QD, 48 weeks (6 cycles) of 28 day on/off via nebulizer (PARI LC sprint)
Serisier	2013	Australia, New Zealand	Ciprofloxacin: 70 (5.6) Placebo: 59.5 (13.2)	RDBPCT, multicenter	42	Ciprofloxacin	Placebo	6 mL (liposome ciprofloxacin 150 mg + free ciprofloxacin 60 mg)	QD, 24 weeks (3 cycles) of 28 days on/off via nebulizer (PARI LC sprint)
Orriols	2015	Spain	Tobramycin: 69.3 (2.1) Placebo: 70.1 (1.9)	RSBPCT, single-center	35	Tobramycin	Placebo	300 mg	BID, 12 weeks via a jet nebulizer
Bilton	2006	USA, UK	Tobramycin: 61.9 (11.4) Placebo: 63.7 (11.7)	RDBPCT, multicenter	53	Tobramycin	Placebo	300 mg/5 mL + 750 mg	BID, 6 weeks via jet nebulizer (PARI LC PLUS)
Drobnic	2005	Spain	NR	RDBPCT, crossover, single-center	60	Tobramycin	Placebo	8 mL	BID, 48 weeks (2 cycles each of 6 months) via a jet nebulizer
Couch and Barker	2001 /2000	US	NR	RDBPCT, multicenter	74	Tobramycin	Placebo	300 mg	BID, 4 weeks via a jet nebulizer
Barker (AIR-BX1)	2015	Australia, Canada, USA	Aztreonam: 64.2 (12.9) Placebo: 64.9 (12.1)	RDBPCT, multicenter	266	Aztreonam	Placebo	75 mg	TID, 16 weeks (2 cycles) of 28 days on/off via eFlow nebulizer
Barker (AIR-BX2)	2015	Australia, Belgium, Canada, France, Germany, Italy, The Netherlands, Spain, UK, USA	Aztreonam: 63.3 (14.2) Placebo: 62.7 (13.3)	RDBPCT, multicenter	274	Aztreonam	Placebo	75 mg	TID, 16 weeks (2 cycles) of 28 days on/off via eFlow nebulizer
Ailiyaer	2018	China	Amikacin: 57.3 (13) Placebo: 56.5 (10.8)	RCT, open-label, multicenter	152	Amikacin	Placebo	5 mL	BID, 2 weeks via a jet atomizer
TR02-107	2014	Bulgaria, Greece, Hungary, India, Serbia, Ukraine	Amikacin: 49.9 (21.1) Placebo: 46.8 (15)	RDBPCT, multicenter	62	Amikacin	Placebo	280 or 560 mg	QD, 4 weeks via eFlow nebulizer
Haworth	2014	United Kingdom, Russia, Ukraine	Colistin: 58.3 (15.3) Placebo: 60.3 (15.8)	RDBPCT, multicenter	144	Colistin	Placebo	1 million IU	BID, 24 weeks via I-neb AAD system
Murray	2011	UK	* Gentamycin: 58 (53–67) Placebo: 64 (55.7–69)	RSBPCT, single-center	65	Gentamycin	Placebo	80 mg	BID, 48 weeks via a jet nebulizer

* Data reported as median (interquartile range). BID: twice a day; DPI: dry powder inhalers; N: number of patients; NR: not reported; RCT: randomized-controlled trial; QD: once a day; RDBPCT: randomized, double-blind, placebo-controlled trial; RSBPCT: randomized, single-blind, placebo-controlled trial; SD: standard deviation; SVN: Small-Volume Nebulizer; TID: three times a day.

**Table 2:** Comparison between ciprofloxacin administered via dry powder inhaler versus small-volume nebulizer.

	Via DPI (Ciprofloxacin) *n* = 3		Via SVN (Ciprofloxacin) *n* = 3		
Outcomes	Trials	% (*n*/N)	Trials	% (*n*/N)	Risk Ratio (% CI)
Time to first pulmonary exacerbation, days	2	-	5	-	87.05 (34.30; 139.79)
Patients with at least one exacerbation	3	38.9 (266/683)	8	42.4 (358/844)	0.98 (0.74; 1.29)
Change in FEV1%	3	-	3	-	-
Change in SGRQ	3	-	6	-	−7.52 (−13.06; −1.98)
Overall mortality	2	1.7 (11/623)	6	2.1 (14/652)	2.81 (0.39; 20.30)
Hospitalizations	1	3.3 (2/60)	5	8.5 (58/678)	-
Eradication pathogens	3	34.3 (228/663)	6	45 (73/162)	0.29 (0.07; 1.26)
Emergence of new respiratory pathogens	3	5.5 (38/683)	2	36.6 (11/30)	0.58 (0.27; 1.23)
Resistance in overall bacteria isolates	2	20.8 (130/623)	10	33.9 (174/513)	1.26 (0.76; 2.09)
Resistance in *P. aeruginosa* isolates	-	-	6	19.6 (75/382)	3.15 (0.09; 109.40)
Change in bacterial density	1	-	9	-	1.50 (−1.13; 4.13)
Drug-related AE	3	19.9 (136/683)	8	33.8 (283/837)	0.99 (0.65; 1.51)
AE leading to drug discontinuation	3	9.6 (66/683)	10	10 (93/925)	0.69 (0.35; 1.32)
Drug-related serious AE	2	2.1 (7/338)	4	2.9 (15/505)	0.90 (0.11; 7.33)
Bronchospasm	3	3.9 (27/683)	5	3.3 (19/563)	0.52 (0.08; 3.26)

AE: Adverse events; CI: confidence interval; DPI: dry powder inhaler; FEV1: forced expiratory volume in 1 s; SGRQ: St. George’s Respiratory Questionnaire; SVN: Small-Volume Nebulizer.

**Table 3:** Factors to be consider when selecting an aerosol delivery device for antibiotics for patients with non-cystic fibrosis bronchiectasis.

1. Device/drug availability
2. Patient age and the ability to use the selected device correctly
3. Clinical setting
4. Device use with multiple medications
5. Cost and reimbursement
6. Convenience in both outpatient and inpatient settings
7. Physician and patient preference

## Data Availability

The data presented in this study are available on request from the corresponding author.

## Supplementary Materials

The following supporting information can be downloaded at: https://www.mdpi.com/article/10.3390/antibiotics11020275/s1, Figure S1: Result of the quality assessment: (A) “Risk of bias” graph, (B) “Risk of bias” summary, Figure S2: Network and forest plot: (A) number of patients experiencing at least one exacerbation, (B) quality of life, (C) spirometry, (D) sputum bacterial density, (E) new respiratory potential pathogens, (F) emergence of *P. aeruginosa* antimicrobial resistance, (G) mortality, (H) drug-related adverse events, (I) adverse events leading to drug discontinuation, (J) serious adverse events, (K) bronchospasm, Figure S3: Funnel plot of (A) mean time to first exacerbation, (B) number of patients experiencing at least one exacerbation, (C) spirometry, (D) bacterial eradication, (E) new respiratory potential pathogens, (F) sputum bacterial density. The contour lines define the region within which 95% of points would be expected to lie in the absence of both heterogeneity and publication bias. The total overall estimate of the meta-analysis is represented by the vertical line, Table S1: Reasons for exclusion according to full-text, Table S2: Inclusion criteria, definition of frequent exacerbations and percent of *P. aeruginosa* of RCTs included in the meta-analysis, Table S3: Checklist of items to include when reporting a systematic review involving a network meta-analysis, Table S4: Pre-specific outcomes according to exacerbation in NCFB patients, Table S5: List of Terms of the Search Strategy*.

## References

[1] Castellani C., Duff A.J.A., Bell S.C., Heijerman H.G.M., Munck A., Ratjen F., Sermet-Gaudelus I., Southern K.W., Barben J., Flume P.A. (2018). ECFS Best Practice Guidelines: The 2018 Revision. J. Cyst. Fibros..

[2] Hill A.T., Sullivan A.L., Chalmers J.D., De Soyza A., Elborn S.J., Floto A.R., Grillo L., Gruffydd-Jones K., Harvey A., Haworth C.S. (2019). British Thoracic Society Guideline for Bronchiectasis in Adults. Thorax.

[3] Martínez-García M.Á., Máiz L., Olveira C., Girón R.M., de la Rosa D., Blanco M., Cantón R., Vendrell M., Polverino E., de Gracia J. (2018). Spanish Guidelines on Treatment of Bronchiectasis in Adults. Arch. Bronconeumol..

[4] Polverino E., Goeminne P.C., McDonnell M.J., Aliberti S., Marshall S.E., Loebinger M.R., Murris M., Cantón R., Torres A., Dimakou K. (2017). European Respiratory Society Guidelines for the Management of Adult Bronchiectasis. Eur. Respir. J..

[5] Chang A.B., Bell S.C., Torzillo P.J., King P.T., Maguire G.P., Byrnes C.A., Holland A.E., O’Mara P., Grimwood K. (2015). Extended voting group Chronic Suppurative Lung Disease and Bronchiectasis in Children and Adults in Australia and New Zealand Thoracic Society of Australia and New Zealand Guidelines. Med. J. Aust..

[6] Tejada S., Campogiani L., Solé-Lleonart C., Gómez A., Gallego M., Vendrell M., Soriano J.B., Rello J. (2021). Inhaled Antibiotics for Treatment of Adults with Non-Cystic Fibrosis Bronchiectasis: A Systematic Review and Meta-Analysis. Eur. J. Intern. Med..

[7] Martínez-García M.Á., Oscullo G., Barreiro E., Cuenca S., Cervera A., Padilla-Galo A., de la Rosa D., Navarro A., Giron R., Carbonero F. (2020). Inhaled Dry Powder Antibiotics in Patients with Non-Cystic Fibrosis Bronchiectasis: Efficacy and Safety in a Real-Life Study. J. Clin. Med..

[8] Rau J.L. (2005). The Inhalation of Drugs: Advantages and Problems. Respir Care.

[9] Dolovich M.B., Ahrens R.C., Hess D.R., Anderson P., Dhand R., Rau J.L., Smaldone G.C., Guyatt G., American College of Chest Physicians (2005). American College of Asthma, Allergy, and Immunology Device Selection and Outcomes of Aerosol Therapy: Evidence-Based Guidelines: American College of Chest Physicians/American College of Asthma, Allergy, and Immunology. Chest.

[10] Rello J., Rouby J.J., Sole-Lleonart C., Chastre J., Blot S., Luyt C.E., Riera J., Vos M.C., Monsel A., Dhanani J. (2017). Key Considerations on Nebulization of Antimicrobial Agents to Mechanically Ventilated Patients. Clin. Microbiol. Infect..

[11] Laska I.F., Crichton M.L., Shoemark A., Chalmers J.D. (2019). The Efficacy and Safety of Inhaled Antibiotics for the Treatment of Bronchiectasis in Adults: A Systematic Review and Meta-Analysis. Lancet Respir. Med..

[12] Xu M.-J., Dai B. (2020). Inhaled Antibiotics Therapy for Stable Non-Cystic Fibrosis Bronchiectasis: A Meta-Analysis. Ther. Adv. Respir. Dis..

[13] Brodt A.M., Stovold E., Zhang L. (2014). Inhaled Antibiotics for Stable Non-Cystic Fibrosis Bronchiectasis: A Systematic Review. Eur. Respir. J..

[14] Uttley L., Tappenden P. (2014). Dry Powder Inhalers in Cystic Fibrosis: Same Old Drugs but Different Benefits?. Curr. Opin. Pulm. Med..

[15] Baez-Pravia O.V., Montes-Andujar L., Menéndez J., Cardinal-Fernández P. (2019). What Have We Learned from Network Meta-Analyses Applied to Critical Care?. Minerva Anestesiol..

[16] De Soyza A., Aksamit T., Bandel T.-J., Criollo M., Elborn J.S., Operschall E., Polverino E., Roth K., Winthrop K.L., Wilson R. (2018). RESPIRE 1: A Phase III Placebo-Controlled Randomised Trial of Ciprofloxacin Dry Powder for Inhalation in Non-Cystic Fibrosis Bronchiectasis. Eur. Respir. J..

[17] Aksamit T., De Soyza A., Bandel T.-J., Criollo M., Elborn J.S., Operschall E., Polverino E., Roth K., Winthrop K.L., Wilson R. (2018). RESPIRE 2: A Phase III Placebo-Controlled Randomised Trial of Ciprofloxacin Dry Powder for Inhalation in Non-Cystic Fibrosis Bronchiectasis. Eur. Respir. J..

[18] Wilson R., Welte T., Polverino E., De Soyza A., Greville H., O’Donnell A., Alder J., Reimnitz P., Hampel B. (2013). Ciprofloxacin Dry Powder for Inhalation in Non-Cystic Fibrosis Bronchiectasis: A Phase II Randomised Study. Eur. Respir. J..

[19] Haworth C.S., Bilton D., Chalmers J.D., Davis A.M., Froehlich J., Gonda I., Thompson B., Wanner A., O’Donnell A.E. (2019). Inhaled Liposomal Ciprofloxacin in Patients with Non-Cystic Fibrosis Bronchiectasis and Chronic Lung Infection with Pseudomonas Aeruginosa (ORBIT-3 and ORBIT-4): Two Phase 3, Randomised Controlled Trials. Lancet Respir. Med..

[20] Ailiyaer Y., Wang X., Zhang Y., Li C., Li T., Qi Q., Li Y. (2018). A Prospective Trial of Nebulized Amikacin in the Treatment of Bronchiectasis Exacerbation. Respiration.

[21] Barker A.F., O’Donnell A.E., Flume P., Thompson P.J., Ruzi J.D., de Gracia J., Boersma W.G., De Soyza A., Shao L., Zhang J. (2014). Aztreonam for Inhalation Solution in Patients with Non-Cystic Fibrosis Bronchiectasis (AIR-BX1 and AIR-BX2): Two Randomised Double-Blind, Placebo-Controlled Phase 3 Trials. Lancet Respir. Med..

[22] Orriols R., Hernando R., Ferrer A., Terradas S., Montoro B. (2015). Eradication Therapy against Pseudomonas Aeruginosa in Non-Cystic Fibrosis Bronchiectasis. Respiration.

[23] Haworth C.S., Foweraker J.E., Wilkinson P., Kenyon R.F., Bilton D. (2014). Inhaled Colistin in Patients with Bronchiectasis and Chronic Pseudomonas Aeruginosa Infection. Am. J. Respir. Crit. Care Med..

[24] Serisier D.J., Bilton D., De Soyza A., Thompson P.J., Kolbe J., Greville H.W., Cipolla D., Bruinenberg P., Gonda I. (2013). ORBIT-2 investigators Inhaled, Dual Release Liposomal Ciprofloxacin in Non-Cystic Fibrosis Bronchiectasis (ORBIT-2): A Randomised, Double-Blind, Placebo-Controlled Trial. Thorax.

[25] Murray M.P., Govan J.R.W., Doherty C.J., Simpson A.J., Wilkinson T.S., Chalmers J.D., Greening A.P., Haslett C., Hill A.T. (2011). A Randomized Controlled Trial of Nebulized Gentamicin in Non-Cystic Fibrosis Bronchiectasis. Am. J. Respir. Crit. Care Med..

[26] Bilton D., Henig N., Morrissey B., Gotfried M. (2006). Addition of Inhaled Tobramycin to Ciprofloxacin for Acute Exacerbations of Pseudomonas Aeruginosa Infection in Adult Bronchiectasis. Chest.

[27] Drobnic M.E., Suñé P., Montoro J.B., Ferrer A., Orriols R. (2005). Inhaled Tobramycin in Non-Cystic Fibrosis Patients with Bronchiectasis and Chronic Bronchial Infection with Pseudomonas Aeruginosa. Ann. Pharmacother..

[28] Couch L.A. (2001). Treatment With Tobramycin Solution for Inhalation in Bronchiectasis Patients with Pseudomonas Aeruginosa. Chest.

[29] Barker A.F., Couch L., Fiel S.B., Gotfried M.H., Ilowite J., Meyer K.C., O’Donnell A., Sahn S.A., Smith L.J., Stewart J.O. (2000). Tobramycin Solution for Inhalation Reduces Sputum Pseudomonas Aeruginosa Density in Bronchiectasis. Am. J. Respir. Crit. Care Med..

[30] Insmed Incorporated (2019). Safety and Tolerability Study of 2 Dose Level of ArikayceTM in Patients with Bronchiectasis and Chronic Infection Due to Pseudomonas Aeruginosa. https://clinicaltrials.gov/ct2/show/NCT00775138.

[31] Tiddens H.A.W.M., Meerburg J.J., van der Eerden M.M., Ciet P. (2020). The Radiological Diagnosis of Bronchiectasis: What’s in a Name?. Eur. Respir. Rev..

[32] Kaplan S., Lee A., Caine N., Charman S.C., Bilton D. (2021). Long-Term Safety Study of Colistimethate Sodium (Colobreathe®): Findings from the UK Cystic Fibrosis Registry. J. Cyst. Fibros..

[33] Konstan M.W., Flume P.A., Kappler M., Chiron R., Higgins M., Brockhaus F., Zhang J., Angyalosi G., He E., Geller D.E. (2011). Safety, Efficacy and Convenience of Tobramycin Inhalation Powder in Cystic Fibrosis Patients: The EAGER Trial. J. Cyst. Fibros..

[34] Solé-Lleonart C., Rouby J.-J., Blot S., Poulakou G., Chastre J., Palmer L.B., Bassetti M., Luyt C.-E., Pereira J.M., Riera J. (2017). Nebulization of Antiinfective Agents in Invasively Mechanically Ventilated Adults: A Systematic Review and Meta-Analysis. Anesthesiology.

[35] Boisson M., Bouglé A., Sole-Lleonart C., Dhanani J., Arvaniti K., Rello J., Rouby J.-J., Mimoz O. (2022). European Investigator Network for Nebulized Antibiotics in Ventilator-Associated Pneumonia (ENAVAP). Nebulized Antibiotics for Healthcare- and Ventilator-Associated Pneumonia. Semin. Respir. Crit. Care Med..

[36] Monsel A., Torres A., Zhu Y., Pugin J., Rello J., Rouby J.-J. (2021). European Investigators Network for Nebulized Antibiotics in Ventilator-associated Pneumonia (ENAVAP) Nebulized Antibiotics for Ventilator-Associated Pneumonia: Methodological Framework for Future Multicenter Randomized Controlled Trials. Curr. Opin. Infect. Dis..

[37] Tejada S., Ramírez-Estrada S., Tejo A.M., Forero C.G., Pomares X., Gallego M., Soriano J.B., Chalmers J.D., Rello J. (2022). Critical Appraisal of International Adult Bronchiectasis Guidelines Using the AGREE II Tool. Eur. J. Intern. Med..

[38] Montón C., Prina E., Pomares X., Cugat J.R., Casabella A., Oliva J.C., Gallego M., Monsó E. (2019). Nebulized Colistin And Continuous Cyclic Azithromycin In Severe COPD Patients With Chronic Bronchial Infection Due To Pseudomonas Aeruginosa: A Retrospective Cohort Study. Int. J. Chron. Obstruct. Pulmon. Dis..

[39] Rello J., Schrenzel J., Tejo A.M. (2021). New Insights into Pneumonia in Patients on Prolonged Mechanical Ventilation: Need for a New Paradigm Addressing Dysbiosis. J. Bras. Pneumol..

[40] Pomares X., Montón C., Bullich M., Cuevas O., Oliva J.C., Gallego M., Monsó E. (2018). Clinical and Safety Outcomes of Long-Term Azithromycin Therapy in Severe COPD Beyond the First Year of Treatment. Chest.

[41] Liberati A., Altman D.G., Tetzlaff J., Mulrow C., Gøtzsche P.C., Ioannidis J.P.A., Clarke M., Devereaux P.J., Kleijnen J., Moher D. (2009). The PRISMA Statement for Reporting Systematic Reviews and Meta-Analyses of Studies That Evaluate Health Care Interventions: Explanation and Elaboration. J. Clin. Epidemiol..

[42] Hutton B., Salanti G., Caldwell D.M., Chaimani A., Schmid C.H., Cameron C., Ioannidis J.P.A., Straus S., Thorlund K., Jansen J.P. (2015). The PRISMA Extension Statement for Reporting of Systematic Reviews Incorporating Network Meta-Analyses of Health Care Interventions: Checklist and Explanations. Ann. Intern. Med..

[43] Higgins J.P.T., Thomas J., Chandler J., Cumpston M., Li T., Page M.J., Welch V.A. (2019). Cochrane Handbook for Systematic Reviews of Interventions.

[44] Dolovich M.B., Dhand R. (2011). Aerosol Drug Delivery: Developments in Device Design and Clinical Use. Lancet.

[45] Wan X., Wang W., Liu J., Tong T. (2014). Estimating the Sample Mean and Standard Deviation from the Sample Size, Median, Range and/or Interquartile Range. BMC Med. Res. Methodol..

[46] Tonin F.S., Borba H.H., Mendes A.M., Wiens A., Fernandez-Llimos F., Pontarolo R. (2019). Description of Network Meta-Analysis Geometry: A Metrics Design Study. PLoS ONE.

