# Supplementary material for: Safety and Efficacy of Devices Delivering Inhaled Antibiotics among Adults with Non-Cystic Fibrosis Bronchiectasis: A Systematic Review and a Network Meta-Analysis
Source: Antibiotics (Basel). 2022 Feb 19;11(2):275. doi: 10.3390/antibiotics11020275 (PMC8868526; doi:10.3390/antibiotics11020275)
Supplement: Supplementary file 1 [file antibiotics-11-00275-s001.zip › antibiotics-1552648-supplementary.pdf]

Table S1. Reasons for exclusion according to full-text.

Reason of exclusion	Study
Not randomized-controlled trials	Loebinger et al., 2019 (trial protocol)<br>López-Gil Otero et al., 2019 (case report)<br>Tran et al., 2019 (experimental study)<br>Burguera-Àvila et al., 2019 (case series retrospective)<br>Berra et al., 2017 (review)<br>Henkle et al., 2017 (survey)<br>White et al., 2012 (case series retrospective)<br>Dhar et al., 2010 (letter)<br>McLeod et al., 2009 (experimental study)<br>Steinford et al., 2007 (brief communication)
Different Comparator	McCullough et al., 2014 (other respiratory medicines such as inhaled corticosteroids or oral azithromycin)<br>Tabernero et al., 2015 (conventional therapy as standardized education and an exercise plan with respiratory physiotherapy)
Different Intervention	Blanco-Aparicio et al., 2019 (intravenous antibiotic vs. oral antibiotic)
Results from other included study	DeVanter et al., 2019 (from Haworth et al., 2019)<br>Sibila et al., 2019 (from Barker et al., 2015)
Others	Hoppentocht et al., 2016 (pk and tolerability of administration)<br>Orriols et al., 2015 (no chronic infection)<br>Gulini et al., 2012 (comparison between kind of nebulizers)<br>Chalmers et al., 2012 (intravenous treatment)<br>Berlana et al., 2011 (not non-cystic fibrosis bronchiectasis patients)<br>Garde et al., 2009 (no treatment study)<br>Scheinberg et al., 2005 (no comparison between treatments)<br>El-Hoffy et al., 2003 (article not found)

Table S2. Inclusion criteria, definition of frequent exacerbations and percent of *P. aeruginosa* of randomized-controlled trials included in the meta-analysis.

Study	Participants	Frequent exacerbations	*P. aeruginosa** % (*n*)
	**via DPI**		
deSoyza et al., 2018<br>Aksamit et al., 2018	Adult patients (≥18 years) had NCFB diagnosed by HRCT, in clinical stable phase, a positive sputum culture at screening of pathogens, and had FEV1 ≥ 30% predicted	At least 2 pulmonary exacerbations treated with antibiotics < 12 months	57.3 (563/982)
Wilson et al., 2013	Adult patients (≥18 years) had NCFB diagnosed by HRCT, in clinical stable phase, had stable disease in the preceding 30 days, were able to produce sputum (≥5 mL) that was culture positive for potential respiratory pathogens, and had FEV1 ≥ 15% predicted	At least 2 pulmonary exacerbations treated with antibiotics < 12 months	54 (67/124)
	**via SVN**		
Hawort et al., 2019	Adult patients (≥18 years) had NCFB confirmed by chest CT, in clinical stable phase, had FEV1 > 25% predicted, and had a history of chronic *P. aeruginosa* lung infection as documented by *P. aeruginosa* culture in a sputum or deep-throat swab or bronchoalveolar lavage or bronchoscopic specimen before the screening visit. A positive sputum or deep-throat swab culture for *P. aeruginosa* with at least one isolate non-resistant to ciprofloxacin was required at screening	At least 2 pulmonary exacerbations treated with antibiotics < 12 months	100 (582/582)
Ailiyaer et al., 2018	Adult patients (≥18 years) had NCFB confirmed by HRCT, in clinical stable phase, had positive sputum cultures for *P. aeruginosa*, and had FEV1 ≥ 30%predicted	NR	100 (152/152)
Barker et al., 2015	Adult patients (≥18 years) had NCFB confirmed by CT, in clinical stable phase, had positive sputum culture for Gram-negative organisms, and had FEV1 ≥ 20%predicted	History of positive bronchoscope culture for target Gram-negative organism or treatment of exacerbation (<5 years) with antibiotics with Gram-negative coverage	80.7 (436/540)
Orriols et al., 2015	Adult patients (≥18 years) had NCFB diagnosed by HRCT, in clinical stable phase, isolation of *P. aeruginosa* in sputum, with sweat tests and blood analyses negative for the most frequent mutations of CFU	NR	100 (35/35)
Haworth et al., 2014	Adult patients (≥18 years) had NCFB conformed by HRCT, in clinical stable phase, a positive sputum culture for *P. aeruginosa* at the screening visit, and had FEV1 ≥ 15%predicted	At least 2 pulmonary exacerbations treated with antibiotics < 12 months	100 (144/144)
Serisier et al., 2013	Adult patients (≥18 years) had NCFB diagnosed by HRCT, in clinical stable phase, with *P. aeruginosa* airway infection, and had FEV1 ≥ 25%predicted	At least 2 pulmonary exacerbations treated with antibiotics < 12 months	100 (42/42)
Murray et al., 2011	Adult patients (≥18 years) had NCFB diagnosed by HRCT, in clinical stable phase, had chronically infected sputum, and had FEV1 > 30%predicted	At least 2 exacerbations in the past year	36.9 (24/65)
Bilton et al., 2006	Adult patients (≥18 years) had NCFB diagnosed by CT, in clinical stable phase, and had positive sputum cultures for	NR	100 (53/53)

	P. aeruginosa within the 12 months before the study		
Drobnic et al., 2005	Adult patients (≥18 years) had NCFB diagnosed by HRCT, in clinical stable phase, and had ≥3 positive sputum cultures for *P. aeruginosa* during the 6 months before the study	NR	100 (60/60)
Couch et al., 2001 Barker et al., 2000	Adult patients (≥18 years) had NCFB diagnosed by CT, in clinical stable phase, and had grossly purulent sputum containing *P. aeruginosa* ≥104 CFU g–1	NR	100 (74/74)
TR02-107	Adult patients (≥18 years) had NCFB diagnosed by HRCT, in clinical stable phase, had chronic *P. aeruginosa* airway infection, had SaO2 ≥90% while breathing room air, and were able to produce ≥0.5 g sputum	NR	100 (62/62)

*We defined the presence of *P. aeruginosa* as a positive culture at screening or at baseline, or both. CFU: colony forming unit; CT: computed tomography; DPI: dry powder inhalers; FEV1: forced expiratory volume in 1 second; HRCT: high-resolution computerized tomography; NCFB: non-cystic fibrosis bronchiectasis; NR: not reported; SVN: Small-Volume Nebulizer.

Table S3. Checklist of items to include when reporting a systematic review involving a network meta-analysis.

Section/topic	Item # *	Checklist item †	Reported on page #
**TITLE**			
Title	1	Identify the report as a systematic review *incorporating a network meta-analysis (or related form of meta-analysis).*	1
**ABSTRACT**			
Structured summary	2	Provide a structured summary including, as applicable:<br>Background: main objectives.<br>Methods: data sources; study eligibility criteria, participants, and interventions; study appraisal; and *synthesis methods, such as network meta-analysis.*<br>Results: number of studies and participants identified; summary estimates with corresponding confidence/credible intervals; treatment rankings may also be discussed. *Authors may choose to summarize pairwise comparisons against a chosen treatment included in their analyses for brevity.*<br>Discussion/Conclusions: limitations; conclusions and implications of findings.<br>Other: primary source of funding; systematic review registration number with registry name.	1
**INTRODUCTION**			
Rationale	3	Describe the rationale for the review in the context of what is already known, *including mention of why a network meta-analysis has been conducted.*	2
Objectives	4	Provide an explicit statement of questions being addressed, with reference to participants, interventions, comparisons, outcomes, and study design (PICOS).	2
**METHODS**			
Protocol and registration	5	Indicate whether a review protocol exists and if and where it can be accessed (e.g., Web address); and, if available, provide registration information, including registration number.	10
Eligibility criteria	6	Specify study characteristics (e.g., PICOS, length of follow-up) and report characteristics (e.g., years considered, language, publication status) used as criteria for eligibility, giving rationale. *Clearly describe eligible treatments included in the treatment network, and note whether any have been clustered or merged into the same node (with justification.*	10
Information sources	7	Describe all information sources (e.g., databases with dates of coverage, contact with study authors to identify additional studies) in the search and date last searched.	11
Search	8	Present full electronic search strategy for at least one database, including any limits used, such that it could be repeated.	11
Study selection	9	State the process for selecting studies (i.e., screening, eligibility, included in systematic review, and, if applicable, included in the meta-analysis).	11
Data collection process	10	Describe method of data extraction from reports (e.g., piloted forms, independently, in duplicate) and any processes for obtaining and confirming data from investigators.	11
Data items	11	List and define all variables for which data were sought (e.g., PICOS, funding sources) and any assumptions and simplifications made.	11
Geometry of the network	S1	Describe methods used to explore the geometry of the treatment network under study and potential biases related to it. This should include how the evidence base has been graphically summarized for presentation, and what characteristics were compiled and used to describe the evidence base to readers.	11
Risk of bias within individual studies	12	Describe methods used for assessing risk of bias of individual studies (including specification of whether this was performed at the study or outcome level), and how this information is to be used in any data synthesis.	11
Summary measures	13	State the principal summary measures (e.g., risk ratio, difference in means); *also describe the use of additional summary measures assessed, such as treatment rankings and surface under the*	11

		cumulative ranking curve (SUCRA) values, as well as modified approaches used to present summary findings from meta-analyses.	
Planned methods of analysis	14	Describe the methods of handling data and combining results of studies for each network meta-analysis. This should include, but not be limited to:<br>*Handling of multigroup trials;*<br>*Selection of variance structure;*<br>*Selection of prior distributions in Bayesian analyses; and*<br>*Assessment of model fit.*	12
Assessment of inconsistency	S2	Describe the statistical methods used to evaluate the agreement of direct and indirect evidence in the treatment network(s) studied. Describe efforts taken to address its presence when found.	12
Risk of bias across studies	15	Specify any assessment of risk of bias that may affect the cumulative evidence (e.g., publication bias, selective reporting within studies).	12
Additional analyses	16	Describe methods of additional analyses if performed, indicating which were prespecified. This may include, but not be limited to, the following:<br>*Sensitivity or subgroup analyses;*<br>*Meta-regression analyses;*<br>*Alternative formulations of the treatment network; and*<br>*Use of alternative prior distributions for Bayesian analyses (if applicable).*	12
**RESULTS ‡**			
Study selection	17	Give numbers of studies screened, assessed for eligibility, and included in the review, with reasons for exclusions at each stage, ideally with a flow diagram.	3
Presentation of network structure	S3	Provide a network graph of the included studies to enable visualization of the geometry of the treatment network.	4-8
Summary of network geometry	S4	Provide a brief overview of characteristics of the treatment network. This may include commentary on the abundance of trials and randomized patients for the different interventions and pairwise comparisons in the network, gaps of evidence in the treatment network, and potential biases reflected by the network structure.	4-8
Study characteristics	18	For each study, present characteristics for which data were extracted (e.g., study size, PICOS, follow-up period) and provide the citations.	4
Risk of bias within studies	19	Present data on risk of bias of each study and, if available, any outcome level assessment.	5
Results of individual studies	20	For all outcomes considered (benefits or harms), present, for each study: 1) simple summary data for each intervention group, and 2) effect estimates and confidence intervals. *Modified approaches may be needed to deal with information from larger networks.*	4-8
Synthesis of results	21	Present results of each meta-analysis performed, including confidence/credible intervals. *In larger networks, authors may focus on comparisons versus a particular comparator (e.g., placebo or standard care), with full findings presented in an appendix. League tables and forest plots may be considered to summarize pairwise comparisons.* If additional summary measures were explored (such as treatment rankings), these should also be presented.	4-8
Exploration for inconsistency	S5	Describe results from investigations of inconsistency. This may include such information as measures of model fit to compare consistency and inconsistency models, P values from statistical tests, or summary of inconsistency estimates from different parts of the treatment network.	4-8
Risk of bias across studies	22	Present results of any assessment of risk of bias across studies for the evidence base being studied.	4-8
Additional analysis	23	Give results of additional analyses, if performed (e.g., sensitivity or subgroup analyses, meta-regression analyses, alternative network geometries studied, alternative choice of prior distributions for Bayesian analyses, and so forth).	4-8
**DISCUSSION**			
Summary of evidence	24	Summarize the main findings, including the strength of evidence for each main outcome; consider their relevance to key groups (e.g., health care providers, researchers, and policymakers).	8

Limitations	25	Discuss limitations at study and outcome level (e.g., risk of bias), and at review level (e.g., incomplete retrieval of identified research, reporting bias). *Comment on the validity of the assumptions, such as transitivity and consistency. Comment on any concerns regarding network geometry (e.g., avoidance of certain comparisons).*	10
Conclusions	26	Provide a general interpretation of the results in the context of other evidence, and implications for future research.	12
**FUNDING**			
Funding	27	Describe sources of funding for the systematic review and other support (e.g., supply of data); role of funders for the systematic review. This should also include information regarding whether funding has been received from manufacturers of treatments in the network and/or whether some of the authors are content experts with professional conflicts of interest that could affect use of treatments in the network.	12

* Boldface indicates new items to this checklist. † Text in italics indicates wording specific to reporting of network meta-analyses that has been added to guidance from the PRISMA statement. ‡ Authors may wish to plan for use of appendices to present all relevant information in full detail for items in this section.

Table S4. Pre-specific outcomes according to exacerbation in non-cystic fibrosis bronchiectasis patients.

Outcome	Definition
**Clinical Outcomes**	
Time to first pulmonary exacerbation	Time in days from the first dose of IA to the first occurrence of a clinically defined pulmonary exacerbation event.
Patients with at least one exacerbation	Proportion of patients with at least one exacerbation from the first dose of IA to the first occurrence of a clinically defined pulmonary exacerbation event.
Lung function (spirometry)	FEV_1 is the maximum amount of air you can forcefully exhale in one second. Change in FEV_1 predicted value is calculated from baseline to the end of the treatment.
Change in quality of life (SGRQ score)	In this questionnaire, higher scores indicate a poorer quality of life. Change in SGRQ is calculated from baseline to the end of the treatment.
Overall mortality	Proportion of overall patients who died at the end of the study,
Hospitalizations	Number of patients with hospitalizations for pulmonary exacerbations at the end of the study.
**Microbiological Outcomes**	
Bacterial eradication	The bacterial eradication rate of the sputum defined as the number of *P. aeruginosa* negative cultured according to sputum culture testing result at the end of treatment (only results from valid cultures that were positive at baseline were considered).
Emergence of new respiratory potential pathogens	The proportion of patients with new pathogenic bacteria occurrence at the end of the treatment period.
Emergence of resistance in total bacterial isolates	The proportion of patients with an increase of the proportion of total bacterial isolates in MIC to IA of > 4 mg/L by susceptibility test at the end of the treatment period.
Emergence of resistance in *P. aeruginosa* isolates	The proportion of patients with an increase of the proportion of *P. aeruginosa* isolates in MIC to IA of > 4 mg/L by susceptibility test at the end of the treatment period.
Bacterial density	Change in bacterial density was measured as the reduction in $\log_{10}$ CFU/g of sputum at the end of the treatment period.
**Safety Outcomes**	
Adverse events	The proportion of patients with drug-related adverse events at the end of the study.
Adverse events leading to drug discontinuation	The proportion of patients who discontinued the administration of the drug due to the occurrence of adverse events.
Serious adverse events	The proportion of patients with drug-related serious adverse events at the end of the study.
Bronchospasm	The proportion of patients with bronchospasm at the end of the study.

CFU: colony forming unit; IA: inhaled antibiotics; FEV1: forced expiratory volume in 1 s; MIC: minimum inhibitory concentration; SGRQ: St George's Respiratory Questionnaire.

Table S5. List of Terms of the Search Strategy*.

#1	"Aerosols" (Mesh)
#2	"Nebulizers and Vaporizers" (Mesh)
#3	nebul* (tiab)
#4	aerosol* (tiab)
#5	vaporiz* (tiab)
#6	inhal* (tiab)
#7	#1 OR #2 OR #3 OR #4 OR #5 OR #6
#8	"Bronchiectasis" (Mesh)
#9	bronchiectasis (tiab)
#10	bacterial respiratory infection* (tiab)
#11	lung infect* (tiab)
#12	non-cystic fibrosis* (tiab)
#13	non-CF* (tiab)
#14	NCFB (tiab)
#15	#8 OR #9 OR #10 OR #11 OR #12 OR #13 OR #14
#16	"Anti-bacterial Agents" (Mesh)
#17	Antimicrobial* (tiab)
#18	anti-microbial* (tiab)
#19	Antibacterial* (tiab)
#20	anti-bacterial* (tiab)
#21	antibiotic* (tiab)
#22	bacterio* (tiab)
#23	#16 OR #17 OR #18 OR #19 OR #20 OR #21 OR #22
#24	colistin* (tiab)
#25	polymyxin* (tiab)
#26	aminoglycoside* (tiab)
#27	amikacin* (tiab)
#28	gentamicin* (tiab)
#29	tobramycin* (tiab)
#30	quinolones* (tiab)
#31	ciprofloxacin* (tiab)
#32	levofloxacin* (tiab)
#33	Beta-lactam* (tiab)
#34	aztreonam* (tiab)
#35	ceftazidime* (tiab)
#36	cefepime* (tiab)
#37	#24 OR #25 OR #26 OR #27 OR #28 OR #29 OR #30 OR #31 OR #32 OR #33 OR #34 OR #35 OR #36
#38	"Pseudomonas" (Mesh)
#39	"Pseudomonas infections" (Mesh)
#40	Pseudomonas aeruginosa (tiab)
#41	P. aeruginosa (tiab)
#42	#38 OR #39 OR #40 OR #41
#43	#23 OR #37
#44	#7 AND #15 AND #42 AND #43

*After some preliminary exercise to test the "Pseudomonas" term, it was added to the search strategy because we tried to maximize sensitivity whilst striving for reasonable precision.

A.

B.

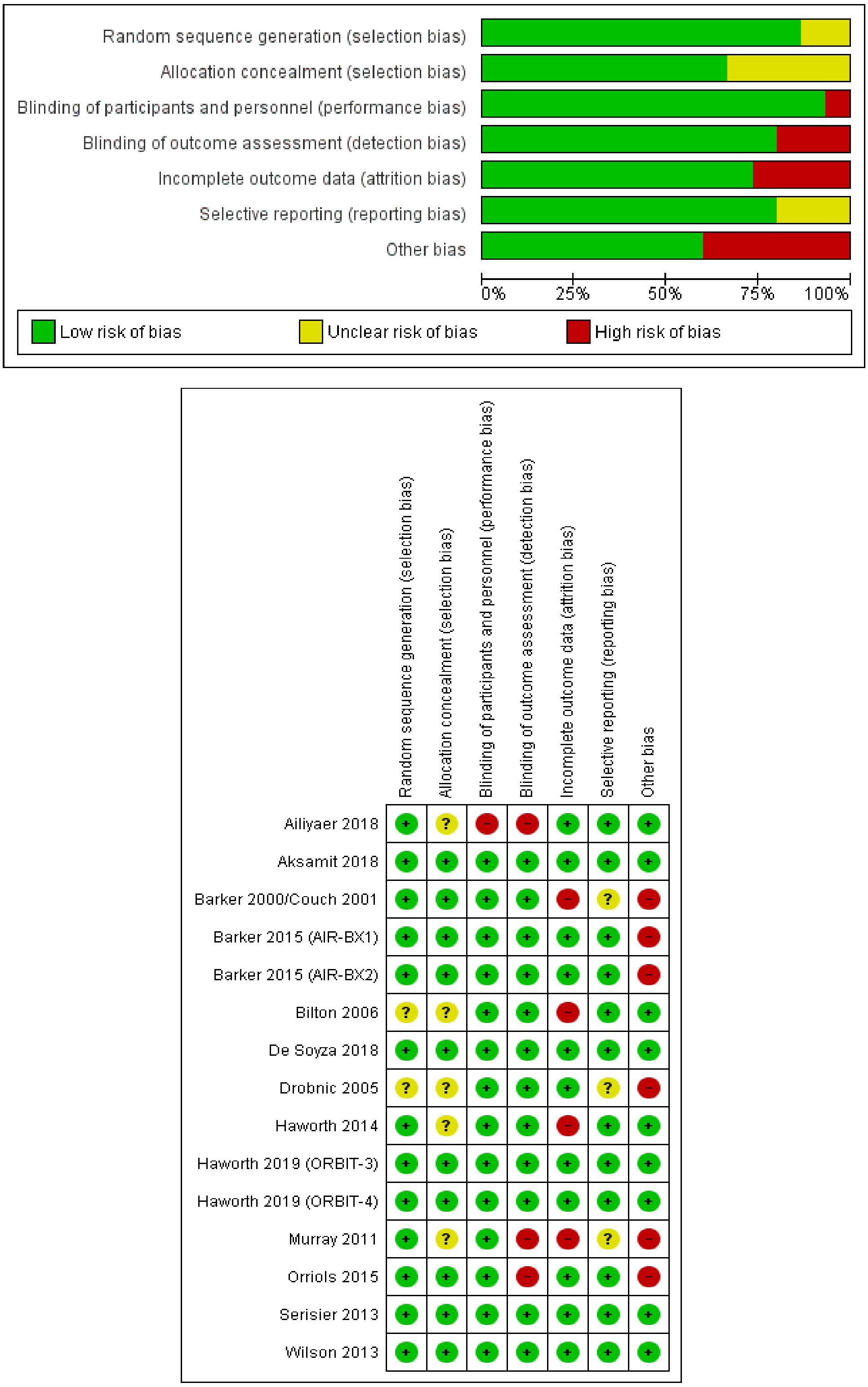

Figure S1. Result of the quality assessment: (A) "Risk of bias" graph, (B) "Risk of bias" summary.

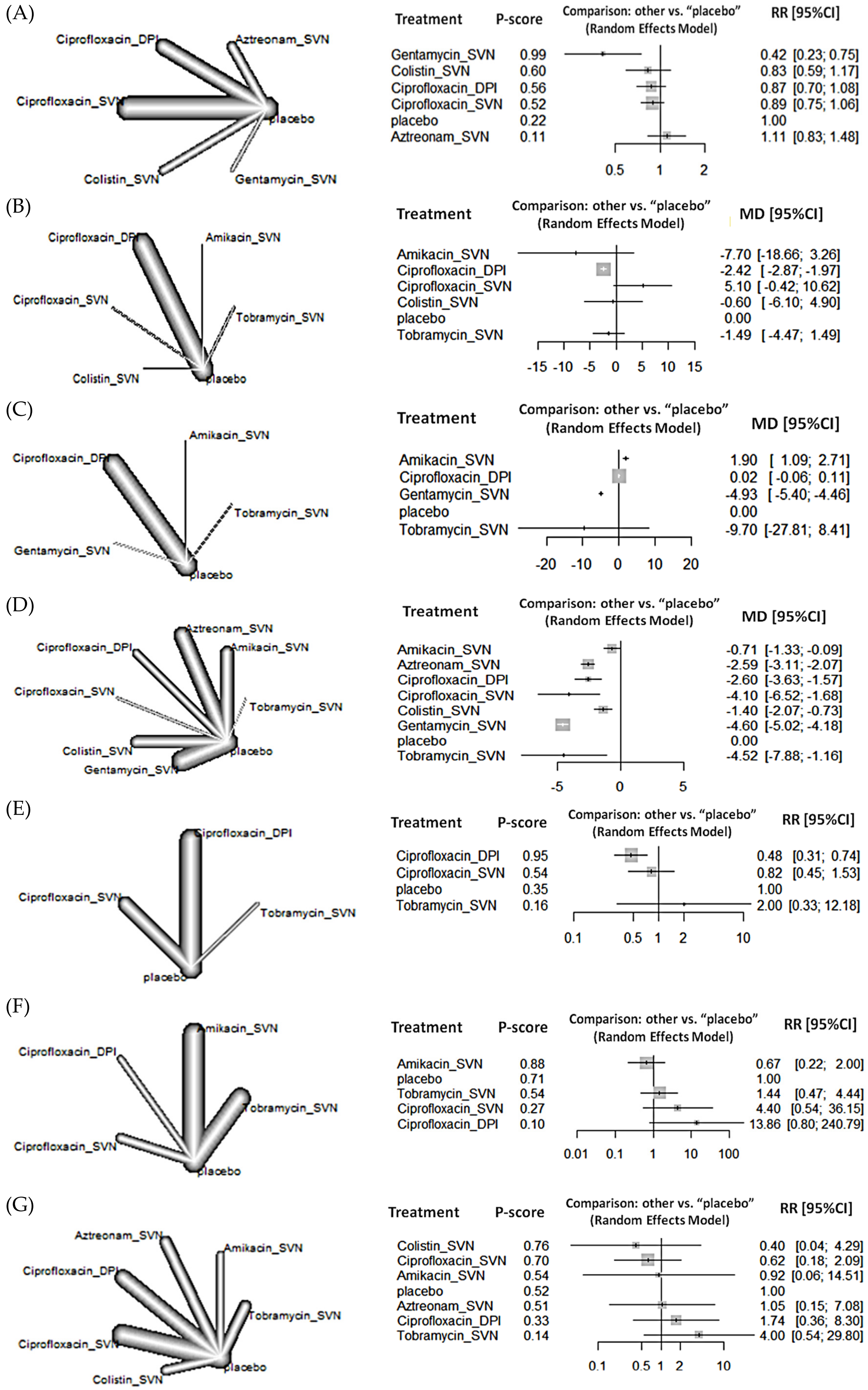
(A)
Ciprofloxacin_DPI
Aztreonam_SVN
Ciprofloxacin_SVN
placebo
Colistin_SVN
Gentamycin_SVN
Treatment
P-score
Comparison: other vs. "placebo"
(Random Effects Model)
RR [95%CI]
Gentamycin_SVN 0.99 0.42 [0.23; 0.75]
Colistin_SVN 0.60 0.83 [0.59; 1.17]
Ciprofloxacin_DPI 0.56 0.87 [0.70; 1.08]
Ciprofloxacin_SVN 0.52 0.89 [0.75; 1.06]
placebo 0.22 1.00
Aztreonam_SVN 0.11 1.11 [0.83; 1.48]
0.5 1 2
(B)
Ciprofloxacin_DPI
Amikacin_SVN
Ciprofloxacin_SVN
Tobramycin_SVN
Colistin_SVN
placebo
Treatment
Comparison: other vs. "placebo"
(Random Effects Model)
MD [95%CI]
Amikacin_SVN -7.70 [-18.66; 3.26]
Ciprofloxacin_DPI -2.42 [-2.87; -1.97]
Ciprofloxacin_SVN 5.10 [-0.42; 10.62]
Colistin_SVN -0.60 [-6.10; 4.90]
placebo 0.00
Tobramycin_SVN -1.49 [-4.47; 1.49]
-15 -10 -5 0 5 10 15
(C)
Amikacin_SVN
Ciprofloxacin_DPI
Tobramycin_SVN
Gentamycin_SVN
placebo
Treatment
Comparison: other vs. "placebo"
(Random Effects Model)
MD [95%CI]
Amikacin_SVN 1.90 [1.09; 2.71]
Ciprofloxacin_DPI 0.02 [-0.06; 0.11]
Gentamycin_SVN -4.93 [-5.40; -4.46]
placebo 0.00
Tobramycin_SVN -9.70 [-27.81; 8.41]
-20 -10 0 10 20
(D)
Aztreonam_SVN
Ciprofloxacin_DPI
Amikacin_SVN
Ciprofloxacin_SVN
Tobramycin_SVN
Colistin_SVN
placebo
Gentamycin_SVN
Treatment
Comparison: other vs. "placebo"
(Random Effects Model)
MD [95%CI]
Amikacin_SVN -0.71 [-1.33; -0.09]
Aztreonam_SVN -2.59 [-3.11; -2.07]
Ciprofloxacin_DPI -2.60 [-3.63; -1.57]
Ciprofloxacin_SVN -4.10 [-6.52; -1.68]
Colistin_SVN -1.40 [-2.07; -0.73]
Gentamycin_SVN -4.60 [-5.02; -4.18]
placebo 0.00
Tobramycin_SVN -4.52 [-7.88; -1.16]
-5 0 5
(E)
Ciprofloxacin_DPI
Ciprofloxacin_SVN
Tobramycin_SVN
placebo
Treatment
P-score
Comparison: other vs. "placebo"
(Random Effects Model)
RR [95%CI]
Ciprofloxacin_DPI 0.95 0.48 [0.31; 0.74]
Ciprofloxacin_SVN 0.54 0.82 [0.45; 1.53]
placebo 0.35 1.00
Tobramycin_SVN 0.16 2.00 [0.33; 12.18]
0.1 0.5 1 2 10
(F)
Amikacin_SVN
Ciprofloxacin_DPI
Tobramycin_SVN
Ciprofloxacin_SVN
placebo
Treatment
P-score
Comparison: other vs. "placebo"
(Random Effects Model)
RR [95%CI]
Amikacin_SVN 0.88 0.67 [0.22; 2.00]
placebo 0.71 1.00
Tobramycin_SVN 0.54 1.44 [0.47; 4.44]
Ciprofloxacin_SVN 0.27 4.40 [0.54; 36.15]
Ciprofloxacin_DPI 0.10 13.86 [0.80; 240.79]
0.01 0.1 1 10 100
(G)
Aztreonam_SVN
Amikacin_SVN
Ciprofloxacin_DPI
Tobramycin_SVN
Ciprofloxacin_SVN
placebo
Colistin_SVN
Treatment
P-score
Comparison: other vs. "placebo"
(Random Effects Model)
RR [95%CI]
Colistin_SVN 0.76 0.40 [0.04; 4.29]
Ciprofloxacin_SVN 0.70 0.62 [0.18; 2.09]
Amikacin_SVN 0.54 0.92 [0.06; 14.51]
placebo 0.52 1.00
Aztreonam_SVN 0.51 1.05 [0.15; 7.08]
Ciprofloxacin_DPI 0.33 1.74 [0.36; 8.30]
Tobramycin_SVN 0.14 4.00 [0.54; 29.80]
0.1 0.5 1 2 10

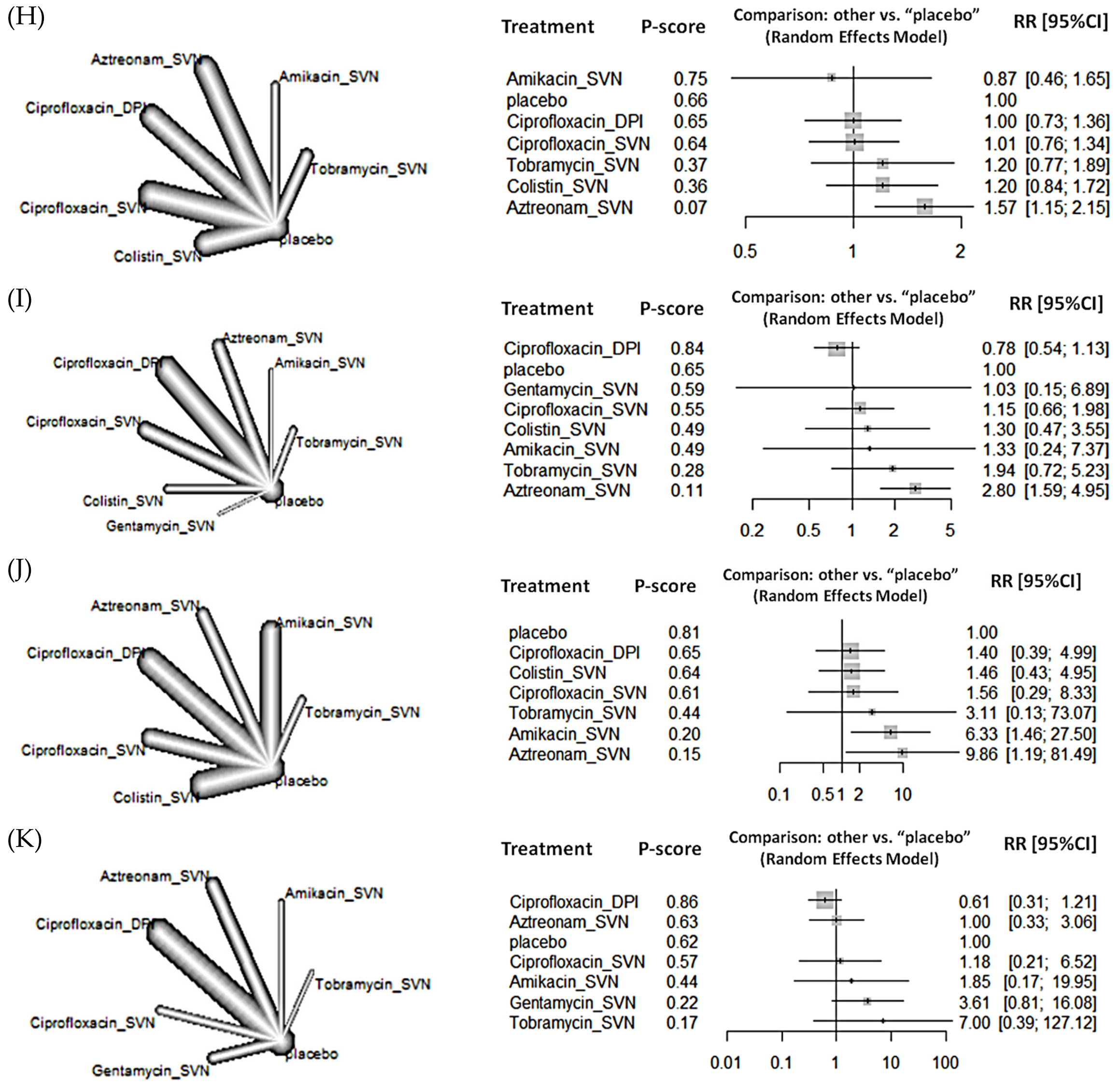

Figure S2. Network and forest plot: (A) number of patients experiencing at least one exacerbation, (B) quality of life, (C) spirometry, (D) sputum bacterial density, (E) new respiratory potential pathogens, (F) emergence of *P. aeruginosa* antimicrobial resistance, (G) mortality, (H) drug-related adverse events, (I) adverse events leading to drug discontinuation, (J) serious adverse events, (K) bronchospasm.

(A)

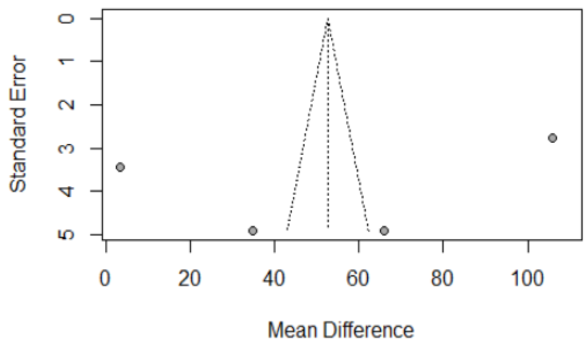
Standard Error
0
1
2
3
4
5
0
20
40
60
80
100
Mean Difference

(B)

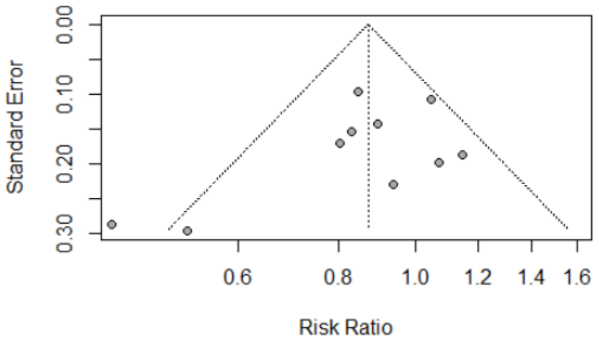
Standard Error
0.00
0.10
0.20
0.30
0.6
0.8
1.0
1.2
1.4
1.6
Risk Ratio

(C)

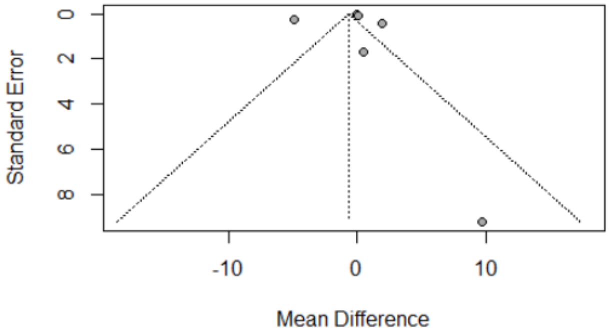
Standard Error
0
2
4
6
8
-10
0
10
Mean Difference

(D)

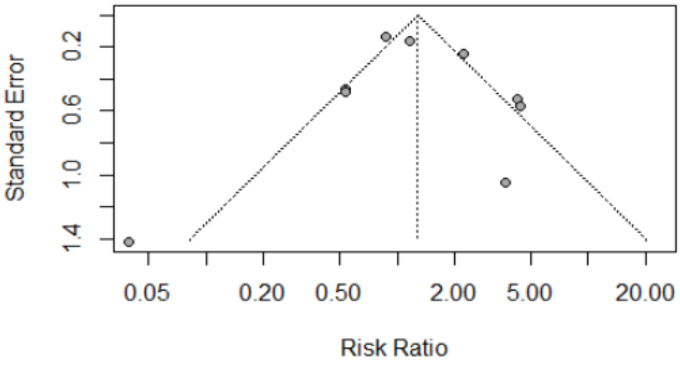
Standard Error
0.2
0.6
1.0
1.4
0.05
0.20
0.50
2.00
5.00
20.00
Risk Ratio

(E)

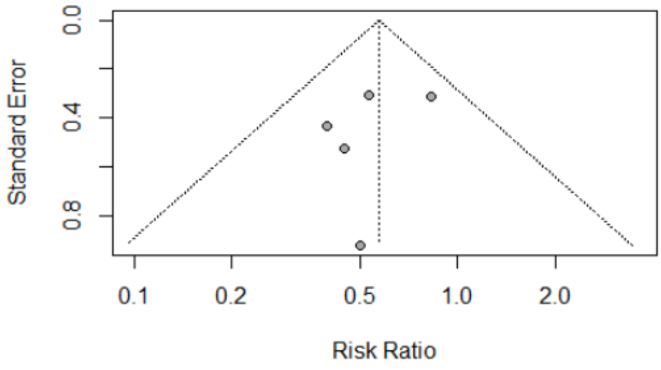
Standard Error
0.0
0.4
0.8
0.1
0.2
0.5
1.0
2.0
Risk Ratio

(F)

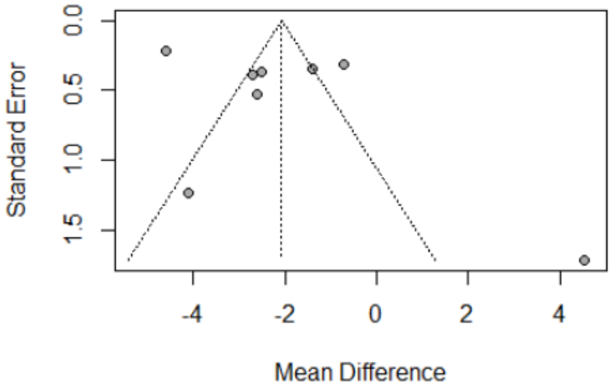

Figure S3. Funnel plot of (A) mean time to first exacerbation, (B) number of patients experiencing at least one exacerbation, (C) spirometry, (D) bacterial eradication, (E) new respiratory potential pathogens, (F) sputum bacterial density. The contour lines define the region within which 95% of points would be expected to lie in the absence of both heterogeneity and publication bias. The total overall estimate of the meta-analysis is represented by the vertical line.
